# Advances in Stem Cell Therapy for Huntington’s Disease: A Comprehensive Literature Review

**DOI:** 10.3390/cells14010042

**Published:** 2025-01-03

**Authors:** Siddharth Shah, Hadeel M. Mansour, Brandon Lucke-Wold

**Affiliations:** Department of Neurosurgery, University of Florida, Gainesville, FL 32608, USAbrandon.lucke-wold@neurosurgery.ufl.edu (B.L.-W.)

**Keywords:** stem cells, Huntington, neurosurgery, neurology, neurodegeneration

## Abstract

Huntington’s disease (HD) is an inherited neurodegenerative disease characterized by uncontrolled movements, emotional disturbances, and progressive cognitive impairment. It is estimated to affect 4.3 to 10.6 per 100,000 people worldwide, and the mean prevalence rate among all published studies, reviews, and genetic HD registries is 5.7 per 100,000. A key feature of HD is the loss of striatal neurons and cortical atrophy. Although there is no cure at present, the discovery of the gene causing HD has brought us into a new DNA era and therapeutic advances for several neurological disorders. PubMed was systematically searched using three search strings: ‘“Huntington disease” + “stem cell”’, ‘”Huntington disease” + Mesenchymal stromal cell’, and ‘”Huntington disease” + “induced pluripotent stem cell”’. For each string, the search results were categorized based on cell type, and papers that included a clinical analysis were categorized as well. The data were extracted up to 2024. We did not include other databases in our search to have a comparable and systematic review of the literature on the topic. The collected data were analyzed and used for critical interpretation in the present review. Data are presented chronologically as clinical studies were published. Therapeutic strategies based on stem cells have drawn a lot of interest as possible HD therapies. Recent research indicates that NSCs have been the most often utilized stem cell type for treating HD. NSCs have been generated and extracted from a variety of sources, including HD patients’ somatic cells and the brain itself. There is strong evidence supporting the transplantation of stem cells or their derivatives in HD animal models, even if stem-cell-based preclinical and clinical trials are still in their early stages. Current treatment only aims at relieving the symptoms rather than treating the pathogenesis of the disease. Although preclinical trials in HD models have shown promise in improving cognitive and motor functions, stem cell therapy still faces many challenges and disadvantages including immunosuppression and immunorejection as well as ethical, technical, and safety concerns. Further research is required for a definitive conclusion.

## 1. Introduction

Huntington’s disease (HD) is a rare neuro-progressive inherited disorder caused by an autosomal dominant mutation in the gene of the protein called huntingtin (Htt). This mutation is the repetition of the DNA main building blocks cytosine, adenine, and guanine (CAG), leading to neuronal cell death and degeneration mainly in the caudate nucleus, putamen, and the cerebral cortex [1,2]. Most people have 27 CAG repeats, not putting them at risk of having HD. However, people with more than 36 –39 CAG repeats people may or may not go on to develop the disease; with 40+ repeats, people will almost certainly go on to develop the disease [3]. For a parent with HD, there is a 50% chance that their offspring will inherit chromosome 4 that carries the HD gene mutation. HD is characterized by motor dysfunction such as chorea, cognitive decline, and psychiatric manifestations such as OCD, bipolar disorder, and depression, hindering normal daily activities [3]. Stem cell therapy has recently been rising as a potential treatment for neurodegenerative disorders such as Alzheimer’s disease (AD), Parkinson’s disease (PD), and Huntington’s disease (HD) for its unique ability to differentiate, regenerate, reverse neuronal damage, and reduce brain inflammation [4]. Recent research has been exploring various types of stem cells including mesenchymal stem cells (MSCs), embryonic stem cells (ESCs), neural stem cells (NSCs), and pluripotent stem cells (iPSC) for their potential to stop HD progression and improve symptoms and quality of life [4,5]. Several preclinical studies have shown the efficacy of stem cell therapy in improving motor function and reversing neuronal damage [6]. Moreover, studies showed that human-derived MSCs offer restoration and protection of the neurons through stem cells’ capability of neural differentiation, neurotrophic support, and antiapoptotic effects [1,2,4,6,7]. With that being said, there are still challenges in stem cell therapy, and to this day, there is still no effective cure for HD, with current treatments only aiming at alleviating the symptoms rather than reversing or stopping neuronal damage, hence the calling for more clinical trials and studies to delve into HD treatment.

This study aims to investigate the advancements and progress made in stem cell therapy, explore its safety and efficacy, and underline its benefits and challenges for its potential future implementation in clinical practice.

## 2. Materials and Methods

This literature review was conducted in PubMed, Embase, and Web of Science. The search terms used for this search were “Huntington disease” + “stem cell”’, ‘”Huntington disease” + Mesenchymal stromal cell’, and ‘”Huntington disease” + “induced pluripotent stem cell”’. For each string, the search results were categorized based on cell type, and papers that included a clinical analysis were categorized as well. The data were extracted up to 2024. We did not include other databases in our search to have a comparable and systematic review of the literature on the topic. The collected data were analyzed and used for critical interpretation in the present review. Data are presented chronologically as clinical studies were published. After this relevant information from each article was entered into an Excel sheet with each section of this manuscript as a separate sheet to ease the process of data extraction for relevant sections.

## 3. Pathophysiology of HD

### 3.1. Oxidative Stress and Mitochondria Dysfunction

In HD etiology, mitochondrial dysfunction (MtD) is a key factor. Striatal degeneration in HD and the pathophysiology of the respiratory chain complex activities are closely related to mitochondrial DNA (mtDNA). In HD brains, there is evidence of abnormal mitochondrial morphogenesis, including increased fission and reduced fusion, as well as mitochondrial loss. HD pathology has been linked to mtDNA loss and reduction in copy number [8]. Even before mutant HTT (mHTT) aggregates form, research suggests that disruption of mitochondrial axonal transport is an important early-stage event in the development of HD [9,10]. The polymorphisms of many SNPs (C16069T, T16126C, T16189C, T16519C, and C16223T), which have been discovered to be able to predict the risk of HD, are mostly due to mutations accruing at a considerably faster rate in the mitochondrial displacement loop (D-loop) than in other areas of mtDNA [11,12]. One of the primary producers of reactive oxygen species (ROS) and nitrogen reactive species (RNS) is mitochondria [13,14]. In HD, mitochondrial dysfunction leads to an overproduction of ROS and a disruption in calcium homeostasis, contributing to oxidative stress (OS) and neuronal injury. Higher amounts of OS have been discovered in HD patients and asymptomatic HD gene carriers, and this is before HD symptoms appear, suggesting that OS is essential to the etiology of the illness [15,16].

### 3.2. HTT and CAG Expansion

Evidence indicates that OS and MtD are the fundamental mechanisms driving the toxicity of mutant HTT (mHTT) in HD pathogenesis, and they also play a significant role in CAG expansion [15,16]. The cytotoxicity caused by mHTT is partly attributable to a change in normal mitochondrial dynamics, which leads to increased mitochondrial fragmentation [17]. HTT is essential for the structure and function of mitochondria [17]. MHTT has been linked to the disruption of proteins that traverse the inner membrane of the mitochondria, which results in neuronal malfunction and death. There is evidence that mHTT causes neuronal dysfunction and cell death in HD brains, impairs mitochondrial function, and interferes with mitochondrial axonal transport [18]. By inducing abnormalities in mitochondrial transcription, decreasing mitochondrial transport to synapses, and directly interacting with mitochondrial architecture in striatal neurons, mHtt increases striatal sensitivity. The binding of mitochondria to valosin-containing protein (VCP) as a result of mHTT is a crucial factor in the neurodegeneration and neuronal death associated with HD [19]. In neurons afflicted by HD, mHTT interacts with mitochondrial fission GTPase dynamin-related protein 1 (Drp1) to cause substantial mitochondrial fragmentation, which leads to aberrant mitochondrial dynamics and neuronal injury. Additionally, it has been discovered that mHTT expression is followed by mtDNA damage and loss, suggesting that mHTT either directly or indirectly compromises mitochondrial function [20]. A higher frequency of mtDNA deletions has been seen in HD patients, suggesting that mHTT and instability of CAG repeats are important contributors to mtDNA damage. In addition to MtD, OS interacts with HTT in HD and contributes to CAG growth. Research conducted on mice and somatic cells has demonstrated a substantial correlation between the degree of CAG repeats and oxidative DNA damage [21]. It has been demonstrated that mHTT increases OS in neuronal and non-neuronal cells, which adds to the pathophysiology of HD. One of the main characteristics of HD is nuclear accumulation of mHTT, and research indicates that OS is essential to this process [22]. The two main characteristics of HD brains are increased OS and striatal susceptibility to mHTT toxicity. Studies have revealed that mHTT expression is connected to alterations in genes, which may result in increased OS and the up-regulation of genes of the antioxidant Nrf2-ARE pathway, possibly in response to elevated levels of OS as a protective mechanism [23]. In cells that express mHTT, OS causes proteasomal dysfunction, which in turn promotes mHTT aggregation and mHTT-induced neuronal death [24]. Furthermore, mHTT results in a decrease in PGC-1α expression and function, which is a co-regulator of mitochondrial biogenesis and antioxidant enzyme production. A PGC-1α deficit increases susceptibility to striatal degeneration and OS [25,26,27,28].

### 3.3. Neurotransmitters

Changes in several neurotransmitters, including acetylcholine, glutamate, GABA, and dopamine (DA), have been implicated in Huntington’s disease (HD) pathology, contributing to its clinical manifestations [29]. Dopamine metabolism has been linked to dysfunction and deletions in mitochondrial DNA (mtDNA), with DA receptors influencing the expression of mitochondrial complex II, a key regulator of mutant huntingtin (mHTT) toxicity. Mitochondria also play a crucial role in the synthesis of neurotransmitters like DA, norepinephrine, GABA, and serotonin [30]. For instance, glutamate synthesis occurs in astrocyte mitochondria, and energy-intensive mitochondrial processes, such as ATP generation, are essential for transporting glutamate from astrocytes to neurons and packaging it into synaptic vesicles [31]. Research indicates that mitochondrial dysfunction (MtD) significantly contributes to glutamate-induced excitotoxicity by impairing neuronal resilience to excitotoxic stress. Glutamate excitotoxicity is a core component of HD etiology and exacerbates mitochondrial Ca2+ overload and cytosolic Ca2+ elevations—hallmarks of excitotoxic damage [32]. Furthermore, GABA-A receptor agonists, known to prevent the loss of mitochondrial membrane potential, exhibit neuroprotective properties. Dysregulation of GABA-A receptors in HD has been associated with increased mitochondrial membrane potential, further promoting neurodegeneration [33].

Glutamate excitotoxicity also induces oxidative stress (OS) due to the failure of antioxidant systems, creating a vicious cycle that disrupts mitochondrial dynamics and exacerbates HD pathology [34]. This cycle involves overexpression of NMDA receptors, increased OS, and further mitochondrial impairment. Notably, GABA plays a protective role by inhibiting OS and maintaining redox balance. In addition, dopamine interacts with mHTT, promoting aggregate formation and activating the proapoptotic transcription factor c-Jun. These effects can be mitigated by interventions such as the c-Jun N-terminal kinase (JNK) inhibitor SP-600125 and the antioxidant ascorbate, which reverse DA-induced mHTT aggregation and c-Jun activation, offering potential therapeutic avenues for HD [35,36,37].

### 3.4. Cytokines and Neuroinflammation

**Figure 1:**
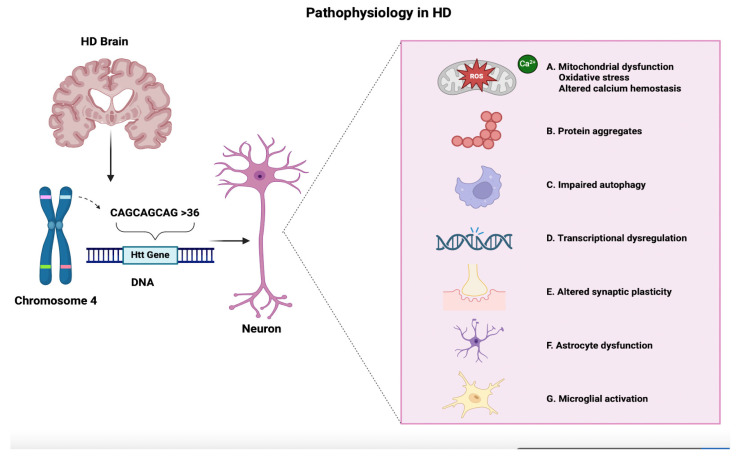
Pathophysiology of Huntington’s disease. This figure illustrates the pathophysiological mechanisms of Huntington’s disease (HD), caused by an expanded CAG repeat (>36) in the huntingtin (Htt) gene on chromosome 4, which results in a mutant huntingtin (mHTT) protein that is toxic to neurons. Several interconnected cellular dysfunctions result from this mutation: (A) Mitochondrial dysfunction and oxidative stress affect energy metabolism and calcium balance, leading to neuronal damage. (B) Protein aggregates of the mutant huntingtin accumulate and disrupt the normal functioning of the cell. (C) Impaired autophagy decreases the cell’s ability to clear damaged components, resulting in toxin accumulation. (D) Transcriptional dysregulation changes the level of gene expression and impacts neuronal function and survival. (E) Altered synaptic plasticity impairs communication between neurons, leading to cognitive and motor dysfunction. (F) Astrocyte dysfunction reduces neuron protection, exacerbating their damage. (G) Microglial activation leads to chronic inflammation and thus enhances neurodegeneration. Altogether, these processes play a role in the progressive decline observed in HD.

Elevated levels of inflammatory mediators in the peripheral and central nervous systems are linked to HD. Studies show that the course of HD is correlated with a reduction in anti-inflammatory cytokine levels and an increase in proinflammatory cytokine levels. Different proinflammatory stimuli can cause microglia activation, which in turn causes the production of pro- and anti-inflammatory cytokines (IL-1β, IL-6, and TNF-α) as well as growth factors (TGF-β, CD206, and Arg1) [38]. A postmortem examination of HD brains revealed a notable accumulation of activated reactive microglia, primarily in the striatum and frontal cortex. Activated microglia are essential to the etiology and development of neurodegenerative disorders [39]. Furthermore, there is a correlation between the number of activated microglia and the degree of HD pathology, indicating a strong association between microglial activation and HD neuronal death [40]. It has been observed that MtD in microglial cells suppresses portions of the IL-4-induced alternative response, which is linked to a reduction in inflammation [41]. This finding raises the possibility that MtD in microglial cells plays a role in proinflammatory mediator expression and neuronal death in neurodegenerative diseases like HD. Additionally, evidence suggests that activated microglia-induced OS may be neurotoxic in part because it causes the generation of reactive oxygen species (ROS), which in turn alters microglial activity and increases the expression of proinflammatory genes [42]. Figure 1 summarizes the pathophysiology of HD.

## 4. Types of Stem Cells, Comparison

The ability of stem cells to divide into distinct adult cell lineages and to self-renew is one of their distinguishing characteristics. Different types of stem cells exist, such as neural stem cells (NSCs), mesenchymal stem cells (MSCs), induced pluripotent stem cells (iPSCs), and embryonic stem cells (ESCs) [43,44]. The variety of potential cell type creation and derivation techniques forms the basis of the categorization. Thus, it is critical to comprehend the traits of the many kinds of stem cells that are now accessible as well as the possible impact of cellular treatments on disease processes. Since each type of stem cell has unique properties and benefits, the justification for using them all depends on the intended applications and results [45,46].

### 4.1. Neural Stem Cells

Compared to ESCs, NSCs in brain tissue are mesenchymal stem cells with more specialization. Neural stem cells (NSCs) exhibit a reduced capacity for self-renewal and often give rise to a restricted range of brain tissue cell lineages, such as neurons, astrocytes, and oligodendrocytes [47,48]. A potential therapeutic option for the treatment of several neurodegenerative illnesses is the transplanting of NSCs to different brain areas. For instance, by producing bioactive chemicals that control synaptic activity, neuronal excitability, and plasticity, NSCs can contribute to gliogenesis. Moreover, NSCs can produce and release antagonistic and synergistic chemicals, which can activate transcription factors, metabolism, and other intracellular NSC regulatory processes [49,50]. Moreover, NSCs can integrate into the current circuitry, form synaptic connections with neighboring neurons, and restore the damaged network. Notably, NSCs are thought to be less carcinogenic and genetically stable than ESCs. By genetically modifying these cells, it is possible to overcome the limited self-renewal potential of NSCs and create immortalized NSCs with increased proliferative capacity [51,52]. However, due to the unavoidable risk of immunological incompatibility in allogeneic transplantation, a lack of sources, challenges in isolating these cells, restricted proliferation and expansion, and ethical and religious concerns, there are still major barriers to the therapeutic application of NSCs [53].

### 4.2. Mesenchymal Stem Cells

Mature and self-renewing mesenchymal stem cells (MSCs) may develop into bone, cartilage, fat, and muscle. Traditionally, MSCs have been discovered in the bone marrow, umbilical cord, adipose tissue, and spleen. Because of their exceptional ability to self-renew while retaining multipotency, MSCs offer a great lot of therapeutic promise and may be the perfect source for cell transplantation in neurodegenerative illnesses [54]. Because MSC-derived functional neurons can be collected more easily than ESCs and there are fewer ethical, religious, and immune rejection issues associated with them, they seem to hold more promise for treating neurodegenerative illnesses. Moreover, unlike other primitive stem cells like ESCs, MSCs do not organize malignancies [55]. Because of their potential, MSCs offer an appealing platform for studying neurodegenerative diseases. Additionally, several studies have suggested that MSCs may be able to pass the blood–brain barrier, which is essential for the appropriate administration of neurotherapeutic drugs into the central nervous system. It has been demonstrated that MSCs can pass through paracellular pathways that would often be blocked by tight junctions to traverse the blood–brain barrier [56]. MSCs are now being used in clinical trials and preclinical research to evaluate the therapeutic efficacy of these cells in a range of neurological illnesses. Intracerebral or intrathecal injections are the two ways that MSCs are administered. After transplantation, MSCs begin to carry out their neuroregenerative duties, which include encouraging endogenous neurogenesis, increasing neuronal development, generating neurotrophic factors, activating microglia, reducing inflammation, and apoptosis and free radical production [57]. Additionally, MSCs can release extracellular matrix elements, angiogenic cytokines, and angiopoietin-1, which enhance angiogenesis and encourage the recruitment of neural progenitor cells (NPCs) [58].

### 4.3. Induced Pluripotent Stem Cells

By inducing the expression of genes and transcription factors that preserve embryonic stem cells, non-pluripotent adult somatic cells—such as fibroblasts, hepatocytes, circulating T lymphocytes, and keratinocytes—can be artificially developed into pluripotent stem cells known as induced pluripotent stem cells (iPSCs) [59]. These reprogrammed cells now offer a viable method for transplanting an infinite number of autologous neurons into individuals suffering from neurodegenerative diseases. An improved differentiation approach can transform iPSCs into full-functioning neuronal lineages, expanding the range of possible applications in the investigation of the processes underlying diverse neurodegenerative illnesses and the identification of new therapeutic targets [52,60]. For instance, a pluripotent cell can be extracted from the skin or blood of a patient suffering from a neurodegenerative illness. The generated iPSCs have the potential to be a dependable source for the generation of degenerative-brain-disease-affected neural cells. Being able to create cells without using oocytes or embryos eliminates ethical and religious concerns, which is one of the key advantages of iPSCs [61,62]. The fact that iPSCs may be created from the patients themselves is another important benefit. This opens up a useful option for autologous cell transplantation without the risk of immunological rejection or the requirement for immunosuppressive medications. Because of their superior specialized terminally differentiated cell phenotypes, easier collection techniques, and lower risk of adverse consequences, iPSCs may have therapeutic benefits [49,63]. Nevertheless, compared to ESCs, IPSC differentiation into adult neurons is more difficult. Similar to ESCs, these cells still carry the danger of developing tumors as a result of unintentional viral integration, which can lead to chromosomal abnormalities, chromosomal disruptions, and low reprogramming effectiveness [64]. Therefore, due to a lack of comprehensive studies testing their therapeutic safety among human patients, the clinical use of IPSCs in neurodegenerative illnesses remains unfeasible [65,66].

### 4.4. Embryonic Stem Cells

A kind of pluripotent stem cells known as embryonic stem cells (ESCs) is obtained from the inner cell mass of blastocysts, which are embryos left to grow for five to six days and have a very sophisticated cellular structure made up of about 100–200 cells. The capacity of embryonic stem cells (ESCs) to self-renew endlessly and specialize into nearly every type of central nervous system cell presents exciting research opportunities [67,68]. These cells are now employed in several neurodegenerative disease research fields as an excellent source of high-purity human neural progenitors in huge quantities. At the moment, ESCs’ therapeutic potential is a major area of research emphasis. Even while ESCs provide novel therapeutic options, the fact that they kill human embryos presents some difficult moral and theological issues [69,70]. Furthermore, all novel ESC therapies in translational medicine are linked to a number of medical concerns, including the high risk of immune rejection in the host patient, tumor formation and cancer due to the persistence of non-differentiated cells undergoing malignant transformation, and genetic instability after an extended period in culture [71,72,73]. Table 1 summarizes the advantages and disadvantages of different types of stem cells.

## 5. Stem Cell Therapy for Huntington’s

The fact that there are currently no effective alternatives to medicine for treating illnesses other than relieving symptoms is a serious issue that affects both the quality of life of patients and caregivers. Therapeutic strategies based on stem cells have drawn a lot of interest as possible HD therapies [74,75]. The replacement of lost or injured neurons carrying enlarged CAG repeats is the main goal of stem cell treatment for Huntington’s disease. There may be new options for the treatment of AD patients with stem cell therapy [76]. An increasing amount of research has demonstrated the effectiveness of neural stem cells (NSCs) derived from embryonic stem cells (ESCs) as a therapeutic method in Alzheimer’s disease (AD) models, demonstrating alterations both in vivo and in vitro. With the help of neurotrophic factors, stem cells may be able to develop into neural cells of the brain and restore neurogenesis and neuroplasticity [77]. Recent research indicates that NSCs have been the most often utilized stem cell type for treating HD. NSCs have been generated and extracted from a variety of sources, including HD patients’ somatic cells and the brain itself. There is strong evidence supporting the transplantation of stem cells or their derivatives in HD animal models, even if stem-cell-based preclinical and clinical trials are still in their early stages [78]. Early stem cell treatments centered on grafting ESC-derived NSCs into HD animals, which showed the integration of motor neurons and host circuit development. Nevertheless, the moral and theological ramifications of fetal material are still important considerations. It is important to take into account the risks associated with stem cell treatments, including graft overgrowth and non-neuronal cells inside grafts [79]. Animal models were used to test the stem cell treatment strategy for AD. In rats with AD, learning and memory were improved by establishing new cholinergic neurons using neural stem cells from their newborn brains [80]. Damaged rat brains were repaired using embryonic stem cells that resembled neurons [81]. Induced pluripotent stem cells (iPSCs), brain-derived neural stem cells (NSCs), mesenchymal stem cells (MSCs), and embryonic stem cells (ESCs) have recently become the most often employed cells in Alzheimer’s disease research [82].

Stem cell therapy for HD is a promising approach that aims to address the root cause of the disease by replacing or repairing damaged neurons and restoring brain function. This contrasts with other therapeutic approaches, such as gene-silencing therapies (e.g., antisense oligonucleotides and RNA interference), which focus on reducing the production of the mutant huntingtin protein to slow disease progression [80]. While gene-silencing therapies target the underlying genetic cause, they do not repair existing neuronal damage or restore lost functions, which is where stem cell therapy could complement these strategies [81]. Small-molecule drugs and symptom-targeting therapies aim to alleviate motor and cognitive symptoms but do not halt or reverse disease progression. In contrast, stem cell therapy offers the potential to regenerate damaged brain tissue, possibly addressing both symptoms and underlying neurodegeneration [82]. However, challenges such as ensuring cell survival, integration, and safety need to be overcome.

Numerous processes have been proposed as being involved in this process, such as reduced beta-amyloid plaques, a sharp decline in β-secretase 1 (BACE-1) levels, decreased tau hyperphosphorylation, the microglia’s reversal of the inflammatory process, and an increase in anti-inflammatory cytokines [83,84,85]. Furthermore, it has been shown that MSCs’ immunomodulation and anti-inflammatory activities work via boosting neuroprotection and reducing proinflammatory cytokines. Furthermore, it has been discovered that bone marrow MSCs promote the development of extracellular and microvesicles. Amyloid-beta is the target of these vesicles in turn. Furthermore, reduced tau phosphorylation, neuroinflammation, tau down-regulation, and developed plasticity and neurotrophic decline are all potential targets for stem cells [86,87]. Transgenic mice demonstrated improved memory and lived with no negative side effects, according to the results. Another study supported this by showing that synaptogenesis enhanced mice’s mental capacity. Encouraging results of successful preliminary animal experiments were obtained. AD mice were injected with human umbilical mesenchymal stem cells taken from donor cords [88,89]. This study concurrently triggered an anti-inflammatory and immune-modulatory response in the animals, and the presence of M2-like microglia increased brain levels of A β and synapsin, which reduced amyloid formation. This resulted in a clinical experiment conducted in 2015 on nine patients with mild-to-to-moderate AD utilizing mesenchymal stem cells (hUCB-MSCs) generated from umbilical cord blood [90,91]. The hippocampal region was stereotactically seeded with hUCB-MSCs. There were no negative effects from the stable and workable stem cell delivery technique. To test the theory, better research with bigger sample sizes and placebo monitoring is required. Although stem cell treatment was administered safely, more research is necessary to determine if it can effectively treat the pathophysiology of AD [92,93].

According to research by Ebert et al. on the brains of mice with Huntington’s disease, mouse-derived NSCs function as GDNF delivery vehicles and help to lessen neuronal loss and the ensuing motor dysfunction [94]. HD animals were given transplants of modified NPCs that overexpress GDNF to investigate the function of environmental enrichment in stem cell treatment. NPCs expressing GDNF provided neuron protection and functional recovery, even though unmodified NSCs exhibited no neuroprotective benefits [94]. Currently, MSCs constitute a viable cell source for HD treatment due to their capacity to lower immune cell malfunction, increase compensatory neurogenesis, lower apoptosis, activate mitochondrial function, and encourage cell survival [95]. Dey et al. (2010) found that in the YAC 128 mouse model of HD, MSCs genetically modified to overexpress BDNF or NGF reduced behavioral impairments and neuronal loss in the striatum [96]. Thus, the striatum may be given a favorable environment to delay neurodegenerative processes by the transplantation of MSCs overexpressing BDNF [96]. Snyder et al. found that the proliferation and neuronal differentiation of endogenous NSCs might be improved by implanting human-derived MSCs into the dentate gyrus of the hippocampus in mouse models of HD [97]. Furthermore, Lin et al. demonstrated that the neuronal differentiation, neurotrophic support capacity, and antiapoptotic actions of human-derived MSCs provided neuroprotection and neurorestoration. As a result, the motor impairment in a mouse model of HD was significantly reduced [7]. Additional research revealed that dental pulp stem cells might be a viable therapeutic source for HD therapy, resulting in decreased immunological rejection following transplantation. Injections of NPCs into the striatum of HD animals also showed comparable functional advantages. These include the integration of NPCs and their migration to secondary locations linked to HD illness, which leads to improvements in function. Two published studies looked at the use of NSCs obtained from iPSCs as a cell replacement therapy for HD [98,99]. Jeon et al. transplanted human-induced pluripotent stem cell (hiPSC)-derived neural stem cells (NSCs) from a patient with 72 CAG repeats into an HD mouse model. The transplanted cells showed no aggregation of human mutant huntingtin (mHTT) and exhibited improved functional outcomes. In contrast, when mouse iPSC-derived NSCs were transplanted into the lateral ventricles of healthy mouse brains, mHTT aggregation was observed in the transplanted cells after 33 weeks. This highlights a difference in mHTT aggregation behavior depending on the origin of the stem cells and the experimental model used [100]. Therefore, it appears that the HD phenotype and permanence of cell death are caused by the autologous transplantation of HD-patient-derived cells with the HD mutation. In the second study, An et al. created human neural stem cells (hNSCs) for transplantation into an HD mouse model by modifying an iPSC mutation acquired from an HD patient [101]. Not only did the transplanted cells survive, but they also effectively developed into motor neurons.

Since nearly all of the research has been conducted on animal models, stem cell therapy is still a long way from being used in a clinical setting to treat HD. More thorough, in-depth preclinical research will be required to verify its therapeutic potential.

### Induced Neuronal Cells

Since it can produce the necessary cells quickly, direct conversion of somatic cells without going through a pluripotency stage is a particularly potent technique for cell therapy. It has been discovered recently that fibroblasts can be directly reprogrammed to become several types of somatic cells, including neural cells. Neural-specific transcription factors have been used to create neural cells from somatic cells, demonstrating that it is feasible to create the necessary cells from many lineages [102,103]. It was initially shown by Vierbuchen et al. that fibroblasts may be directly converted into neural cells by the induction of transcription factors unique to distinct neuronal lineages. When embryonic and postnatal mouse fibroblasts were exposed to three transcription factors (ABM; Ascl1, Brn2, and Myt1l), which were chosen from 19 candidate genes using lentiviral selection, they were instantly transformed into functioning neural cells. The cells were dubbed induced neuronal cells (iNs) because they exhibited characteristics of neurons, an ability to establish synapses and electrical activity [104]. Furthermore, Pang et al. employed the same components that were utilized to transform mouse fibroblasts into human iNs. However, in human fibroblasts, the identical set of conditions did not produce the same result, and the functional characteristics of the transformed neural cells were restricted [105]. After screening 20 more variables in conjunction with ABM, they discovered that co-expressing ABM and the transcription factor NeuroD1, which encodes a basic helix–loop–helix (bHLH), may transform human fibroblasts into viable neural cells. These findings suggest that mouse and human cells may have different levels of direct reprogramming efficacy. Furthermore, it has been documented that microRNA, or miRNA, is crucial for cell reprogramming. It was discovered by two groups that miRNAs function as transcription factors unique to neurons and stimulate the transformation of human fibroblasts into neural cells. Ambasudhan et al. demonstrated that Ascl1 was not necessary for neuronal conversion and that miR-124 could induce iNs when combined with Brn2 and Myt1l [103]. According to Yoo et al., the expression of miR-124 and miR-9/9* in postmitotic neurons has an impact on neuronal conversion [106]. Additionally, it was shown that the creation of neuronal subtypes such as dopaminergic neurons, motor neurons, and GABAergic neurons occurs in mouse and human fibroblasts when tissue-specific transcription factors are expressed [107,108,109,110,111]. Table 2 summarizes the clinical trials on MSCs for HD.

Four clinical trials in Brazil have explored different stem cell therapeutic approaches for Huntington’s disease (HD), a neurodegenerative disorder for which no effective treatments have been found yet. The ADORE-EXT trial is an extension study designed to evaluate the long-term safety and efficacy of the Cellavita-HD product in HD patients who participated in earlier trials [112]. Longitudinal studies like ADORE-EXT are vital for determining whether therapeutic benefits from stem cell therapy can be sustained over extended periods. They also provide crucial insights into long-term adverse effects that may not manifest during initial trial phases. However, a key challenge for such trials lies in ensuring consistent follow-ups, especially as HD patients experience cognitive and physical decline over time. This study is still being performed with 35 participants and aims at assessing the effects of multiple Cellavita-HD administrations, based on MSCs’ possible anti-inflammatory and neuroprotective effects. However, the trial is ongoing, but not recruiting, which suggests that data are being collected to determine the long-term efficacy of the therapy.

Other trials include ADORE-DH and SAVE-DH, which have extended research on the use of Cellavita-HD in HD treatment [113,114]. The final study of ADORE-DH defined the dose–response relationship of Cellavita-HD, which is essential for achieving the highest therapeutic impact and the lowest side effect profile for further research studies such as ADORE-EXT. The SAVE-DH trial assessed the safety of intravenous Cellavita-HD in a smaller male population which helped in the advancement of protocols for MSC-based therapies. Furthermore, the STAR study also evaluates the safety and efficacy of NestaCell®, which is derived from human dental pulp stem cells (hDPSCs) in 120 patients with Huntington’s disease [115]. Even though this is a Phase 3 trial, it is not recruiting, but it investigates another stem cell source that may have better capabilities for regeneration and wider treatment applications. Since all these studies are inconclusive, there is a need for more clinical trials to confirm and explore the possibilities of stem cell therapies in the treatment of HD (Table 2).

Stem cell therapy trials for HD have shown promising but mixed results. While some studies report improvements in motor function and cognitive performance, these findings are often preliminary and require validation in larger, long-term studies. Establishing a consistent success rate is further complicated by the small sample sizes typical of HD trials, a consequence of the disease’s low prevalence. Side effects remain a critical concern in stem cell therapy. Reported adverse events include immune reactions, infections, and complications related to the delivery method, such as intravenous or intrathecal administration. While these risks are generally manageable, their occurrence underscores the need for rigorous safety monitoring and robust patient education. Implementing stem cell therapies in clinical practice presents unique challenges. Standardizing cell product quality, dosage, and delivery methods is essential to ensure consistency and reliability across treatments. Regulatory hurdles, including stringent safety and efficacy requirements, can prolong the development and approval process. Additionally, recruiting participants for HD trials is difficult due to the disease’s rarity and progressive nature. Ethical considerations further complicate matters, particularly regarding consent in advanced disease stages when cognitive decline may impair decision-making capacity.

## 6. Discussion and Future Direction

The increase in CAG repeats at the Htt gene, which causes the loss of GABAergic MSNs in the striatum, is the cause of HD, a hereditary neurodegenerative disease marked by motor and cognitive impairments. The creation of a cell transplantation therapy using induced neural stem cells (iNSCs) is enticingly viewed as a novel therapeutic approach for HD treatment, as no medication can completely treat HD [76]. Direct conversion technology from somatic cells to NSCs has recently been created, and the iNSCs maintain the capacity to differentiate into any neuronal lineage while also being able to self-renew. However, they were created primarily from nonhuman cells through a variety of delivery techniques, including small molecules, growth factors, and viral or nonviral plasmid systems containing foreign DNA. This could lead to genomic instability and, as a result, reduce the likelihood of HD treatment being used in clinical settings in the future [116]. Therefore, for complete reprogramming of neural cell types, the iNSCs must be efficiently generated from human cell sources, ideally as close to the human brain’s original cell as possible, using small molecules or other DNA-free systems. After transplantation, the iNSCs must then differentiate into functional GABAergic MSNs that do not form tumors in HD patients. Furthermore, it has been discovered that iNSCs may have therapeutic cells for clinical applications by transplantation into the brains of disease model animals and behavior recovery [117]. Though the exact mechanisms were not fully understood, it is important to take into account the interactions between the transplanted iNSCs and immune cells because their mode of therapeutic action may depend on cell replacement and immunomodulatory or neuroprotective effects. This is especially true when sufficient numbers of iNSCs are transplanted into the areas of the injured brain. Furthermore, the production of iNSCs from HD patients’ somatic cells is necessary for stem-cell-based therapeutic applications; however, the enlarged CAG repeats in the iNSCs from HD patients need to be replaced with normal-length CAG repeats using gene correction procedures [69].

The CRISPR/Cas9 gene-editing technology offers transformative potential for stem cell therapies in Huntington’s disease (HD) by enabling precise correction of the mutant huntingtin (mHTT) gene in patient-derived stem cells. When combined with human-induced pluripotent stem cells (hiPSCs), CRISPR/Cas9 can be used to edit the expanded CAG repeats in the HTT gene, generating corrected cells that can be differentiated into healthy neurons for transplantation [118]. This approach not only addresses the genetic root cause of HD but also provides an unlimited supply of autologous, genetically corrected cells, minimizing immune rejection risks. It is important to consider the possibility of off-target effects while using the CRISPR/Cas-9 technology, as they might result in unanticipated mutations that could have disastrous repercussions on patients. Thus, in the near future, the CRISPR/Cas9 system’s imprecise technology has to be modified for clinical applications. In conclusion, one of the most promising therapies for neurodegenerative illnesses is cell transplantation therapy using autologous iNSCs directly converted from patients. However, in the next few years, more research should be conducted to determine the benefits of autologous iNSCs for the treatment of HD [118].

Embryonic stem cells (ESCs), neural stem cells (NSCs), and mesenchymal stem cells (MSCs) each hold unique potential for treating Huntington’s disease (HD). ESCs can differentiate into any cell type, including neurons, offering the possibility to replace damaged neurons and restore lost functions in HD [55]. However, their use is limited by ethical concerns and potential risks, such as tumor formation. NSCs, which are more lineage-restricted, can directly replenish neuronal and glial populations in the brain and have shown promise in improving motor and cognitive functions in HD animal models [118]. Additionally, NSCs may secrete neuroprotective factors that support the survival of existing neurons. MSCs, derived from sources like bone marrow or adipose tissue, have a lower tumorigenic risk and are known for their immunomodulatory and neuroprotective properties. MSCs can secrete trophic factors that reduce inflammation, enhance neuroprotection, and promote the repair of damaged neural networks [54]. Combining these stem cell types with advanced genetic engineering or drug delivery systems could further enhance their efficacy in HD therapy, addressing both the symptoms and the underlying disease mechanisms.

## 7. Conclusions

There is no effective treatment for curing Huntington’s Disease (HD) or slowing its progress. Current treatment only aims at relieving the symptoms rather than treating the pathogenesis of the disease. Although preclinical trials in HD models have shown promise in improving cognitive and motor functions, stem cell therapy still faces many challenges and disadvantages including immunosuppression and immunorejection as well as ethical, technical, and safety concerns. Through the use of transcription factors, miRNA, and small compounds, direct conversion technology has been established to produce neural cells and NSCs from somatic cells. While the iNSCs had the potential to develop into neurons, astrocytes, and oligodendrocytes, the iNs were already postmitotic and differentiated.

Emerging research has identified pluripotent stem cells, such as iPSCs, and NSCs as particularly promising due to their ability to differentiate into neurons and integrate into damaged brain circuits. Moreover, advancements in gene-editing technologies like CRISPR/Cas9 hold significant promise for improving stem-cell-based therapies by enabling precise genetic corrections in patient-derived stem cells, reducing the risk of rejection and enhancing treatment specificity. Future research should prioritize optimizing cell delivery methods, ensuring the survival and functional integration of transplanted cells, and addressing safety concerns such as tumor formation or immune responses. Additionally, combining stem cell therapy with other therapeutic approaches, such as gene silencing or neuroprotective agents, could offer synergistic benefits. By focusing on these areas, the field can move closer to developing effective, scalable, and personalized stem cell therapies for HD, offering hope for improved outcomes and quality of life for patients.

Furthermore, it has been discovered that iNSCs may be used as therapeutic cells for clinical applications by being transplanted into the brains of animals model-afflicted with various diseases and leading to behavioral recovery. When combined, the iNSCs extracted from HD patients have enormous promise as a source of therapeutic stem cells for HD therapies on the road. Nevertheless, the enlarged CAG repeats seen in the iNSCs created from the somatic cells of HD patients need to be replaced with normal-length CAG repeats using gene-editing methods like CRISPR/Cas-9. As much as we hope for stem cells to be the new cutting-edge treatment for this devastating and potentially fatal disease, extensive research and clinical trials are required to prove their feasibility and efficacy in reducing neuronal damage, reversing cell death, and stopping cognitive and motor dysfunction in humans.

## 8. Limitations

Although there are still a number of obstacles to overcome, mesenchymal stem cells (MSCs) have the potential to be neuroprotective and may be used to treat Huntington’s disease (HD). The majority of research to date has been conducted on animal models, and as there are no solid findings from human clinical trials, it is unclear if they are effective in treating human patients. The effectiveness and consequences of different types of stem cells can vary, and it is unclear which cell type would yield the greatest results. Furthermore, there are no comprehensive follow-up trials substantiating the long-term benefits of MSCs in HD, and their long-term safety and effectiveness are poorly known. The use of stem cells raises additional safety and feasibility issues for clinical application and ethical questions as well as practical difficulties since delivering stem cells into the brain is extremely difficult.

## Figures and Tables

**Table 1:** Advantages and disadvantages of different types of stem cells.

Type of Stem Cell	Advantages	Disadvantages
NSCs	Low risk of tumor formation	Moral dilemmas, immune rejection risk, limited differentiation, low capability for self-renewal, limited proliferation and growth, and limited availability Challenging isolation techniques
MSCs	No moral dilemmas, excellent reach, simple isolation techniques, generation of autologous cells, self-renewing ability, minimal chance of immunological rejection	Risk of tumor formation
IPSCs	No ethical problems, low risk of immune rejection, high accessibility	High risk of tumor development, risk of recurrence of the patient’s initial pathology, anomalies of the genome, and epigenetics
ESCs	Unlimited proliferation	Ethical issues, immune rejection risk, erratic distinction, elevated chance of tumor development

**Table 2:** MSC clinical trials registered at clinicaltral.gov (accessed 10 August 2024).

Study ID	Title	Status	Country	Participants n=	Sex	Intervention	Phase
NCT04219241 (112)	Clinical Extension Study for Safety and Efficacy Evaluation of Cellavita-HD Administration in Huntington’s Patients. (ADORE-EXT)	Active/not recruiting	Brazil	35	All		Phase 2 Phase 3
NCT03252535 (113)	Dose-response Evaluation of the Cellavita HD Product in Patients With Huntington’s Disease (ADORE-DH)	Completed	Brazil	35	All	Biological: Cellavita-HD	Phase 2 Phase 3
NCT02728115 (114)	Safety Evaluation of Cellavita HD Administered Intravenously in Participants With Huntington’s Disease (SAVE-DH)	Active/not recruiting	Brazil	6	Male		Phase 2 Phase 3
NCT06097780 (115)	Efficacy and Safety of NestaCell® in Huntington’s Disease (STAR)	Not recruiting	Brazil	120	All	Dental Pulp Stem Cells (hDPSCs)	Phase 3

## Data Availability

No new data were created or analyzed in this study.
